# Norwegian reference values for the Short-Form Health Survey 36: development over time

**DOI:** 10.1007/s11136-017-1684-4

**Published:** 2017-08-14

**Authors:** Ellisiv L. Jacobsen, Asta Bye, Nina Aass, Sophie D. Fosså, Kjersti S. Grotmol, Stein Kaasa, Jon Håvard Loge, Torbjørn Moum, Marianne J. Hjermstad

**Affiliations:** 1 Department of Physiotherapy, Faculty of Health Sciences, Oslo and Akershus University College of Applied Sciences, Oslo, Norway; 2 Regional Advisory Unit for Palliative Care, Department of Oncology, Oslo University Hospital, Oslo, Norway; 3 Department of Nursing and Health Promotion, Faculty of Health Sciences, Oslo and Akershus University College of Applied Sciences, Oslo, Norway; 4 Faculty of Medicine, University of Oslo, Oslo, Norway; 5 Department of Oncology, Oslo University Hospital, Oslo, Norway; 6 National Advisory Unit on Late Effects After Cancer Treatment and Department for Clinical Service, Oslo University Hospital, Oslo, Norway; 7 European Palliative Care Research Centre (PRC), Department of Cancer Research and Molecular Medicine, Faculty of Medicine, Norwegian University of Science and Technology (NTNU), Trondheim, Norway; 8 Department of Behavioural Sciences in Medicine, Faculty of Medicine, University of Oslo, Oslo, Norway

**Keywords:** Reference values, Quality of life, HRQoL, SF-36, Stability, General population

## Abstract

**Purpose:**

Reference values for patient-reported outcome measures are useful for interpretation of results from clinical trials. The study aims were to collect Norwegian SF-36 reference values and compare with data from 1996 to 2002.

**Methods:**

In 2015, SF-36 was sent by mail to a representative sample of the population (*N* = 6165). Time trends and associations between background variables and SF-36 scale scores were compared by linear regression models.

**Results:**

The 2015 response rate was 36% (*N* = 2118) versus 67% (*N* = 2323) in 1996 and 56% (*N* = 5241) in 2002. Only 5% of the youngest (18–29 years) and 27% of the oldest (>70 years) responded in 2015. Age and educational level were significantly higher in 2015 relative to 1996/2002 (*p* < .001). The oldest age group in 2015 reported better scores on five of eight scales (*p* < 0.01), the exceptions being bodily pain, vitality, and mental health compared to 1996/2002 (NS). Overall, the SF-36 scores were relatively stable across surveys, controlled for background variables. In general, the most pronounced changes in 2015 were better scores on the role limitations emotional scale (7.4 points, *p* < .001) and lower scores on the bodily pain scale (4.6 points, *p* < .001) than in the 1996/2002 survey.

**Conclusions:**

The low response rate in 2015 suggests that the results, especially among the youngest, should be interpreted with caution. The high response rate among the oldest indicates good representativity for those >70 years. Despite societal changes in Norway the past two decades, HRQoL has remained relatively stable.

## Introduction

Patient-reported outcome measures (PROMs) represent patients’ own perceptions on their health and well-being [1]. This is important as evaluations by health care professionals may differ considerably from the patients’ own perceptions [2–4]. Therefore, PROMs have been recognized as independent outcomes in clinical studies [1] and in health care research in general [2].

PROMs is an umbrella term that includes different dimensions of a person’s health [5] and covers both unidimensional and multidimensional constructs. The latter includes measures of Health-Related Quality of Life (HRQoL) [2, 5]. One of the most widely used HRQoL measures is the Medical Outcome Study 36-item short form (SF-36) [6]. The SF-36 is a generic PROMS tool, i.e., not specific for any population or disease, and assesses HRQOL by eight different scales covering aspects of mental health, physical health, and social functioning [7]. SF-36 has been used in health policy evaluations, clinical practice and research, health interventions, and general population surveying.

Reference data are essential to evaluate whether an individual or a group score is above or below the average for their gender, age, region, country or adjusted for other relevant characteristics. Therefore, reference values for the SF-36 have been developed and published in many countries [8–13]. The first Norwegian reference values for the SF-36 were published in 1996 [14]. In 2002, SF-36 data were also collected from a representative sample of the Norwegian population as part of the Norwegian Level of Living Survey conducted by Statistics Norway [15]. In 2017, one paper was published based on the 2002 survey data, aiming to update the normative data and examining the measurement properties of the Norwegian SF-36 [16]. Since then, to our knowledge, no new reference values have been collected or published for the Norwegian population.

Clinicians and other users might question the validity of comparing the relatively old SF-36 reference values with recent patient data as several demographic and lifestyle changes have occurred in Norway and other Western countries during the last decades [17, 18]. In the same period, the number of expected life years has increased, and overall, the Norwegian population leads healthier lives than before. For instance, the percentage of individuals who never exercised decreased by almost 40% in 2015 compared to 1998 [17]. In addition, the proportion of daily smokers has declined steadily over the past 40 years, with approximately 10% daily smokers in 2015 compared to 33% in 2001 [19]. On the other hand, the prevalence of obesity (BMI ≥ 30) increased from 5% in 1995 to 12% in 2015 [17]. Furthermore, there is currently a higher proportion of immigrants in Norway than when the SF-36 reference values were first obtained [18]. These changes have led to an older and more diverse population and may have introduced a greater difference in health-related behaviors between different socio-economic groups. Additionally, research on the performance and stability of the SF-36 in the general population over time is sparse [20–22]. One study assessing the stability of HRQoL scores in Norway using the European Organisation for Research and Treatment of Cancer Core Quality of Life Questionnaire (EORTC QLQ-C30) showed that scores remained relatively stable over an eight-year period [23]. Still, the increased focus on the patient perspective in clinical studies has led to a request for updated SF-36 scores.

Study objectives were to (1) present new reference values for the SF-36 and (2) examine the stability of SF-36 scores over the past 19 years, controlling for gender, age, and education, by comparing data from the 2015, 1996, and 2002 surveys.

## Materials and methods

### Data collection

The data in this report were obtained from three different surveys counting 9837 randomly drawn respondents who completed surveys in 2015 (*n* = 2118), 1996 (*n* = 2323), and 2002 (*n* = 5396). All subjects received a postal questionnaire including the SF-36, and questions regarding sociodemographic variables. The results from the 1996 and 2002 surveys have been presented in detail previously [14–16], and thus their methods and results will be presented only in brief. In the present study, the stability of HRQoL was investigated by determining the ability of the SF-36 subscales to identify similarities and differences across the three surveys [21].

In 2015, a total of 6165 subjects, aged 18–80 years, who were representative of the general Norwegian population with respect to age, gender, and place of residence, was randomly drawn by Bring Dialog. In the 1996 survey, a representative sample of 3500 subjects aged 19–80 years was randomly drawn by the Norwegian Government Computer Center (SDS) from the National Register [14]. In the 2002 survey, a sample of 10,000 subjects ≥15 years was randomly drawn from Statistics Norway’s database of demographics/the Norwegian population (BEBAS) [15, 16].

### Material

#### The SF-36

The Norwegian version of the SF-36 version 1 was used in all three surveys. This questionnaire consists of 36 items, grouped into eight multi-item scales that measure physical functioning (PF), role limitations due to physical problems (RP), bodily pain (BP), general health (GH), vitality (VT), social functioning (SF), and role limitations due to emotional problems (RE) and mental health (MH) [24]. Item scores were transformed to 0–100 point scales (0 = worst, 100 = best) using the SF-36 algorithm [7]. As per the SF-36 algorithm, single imputation was employed meaning that missing values were replaced with the subjects’ mean score for the completed items on the same scale if more than 50% of the scale’s items were completed [24]. Previous international and Norwegian studies have found SF-36 to be a valid, reliable, and suitable measurement of HRQoL [20, 24–27].

#### Sociodemographic variables

Only variables measured in the same manner in all three surveys are included in the analysis. All subjects were asked about their age, gender, and highest completed level of education. The 2015 survey included a question about the subjects’ living situation, i.e., whether they were living alone, with other adults, or with children younger than 15 years. Education was divided into three groups based on the level of education: second level, first stage (elementary and/or primary school); second level, second stage (high school); and third level (university college or university).

### Statistical analysis

Normally distributed continuous variables are described using the means and standard deviations, while categorical variables are described as percentages. Chi-squared test was used to assess the associations between categorical variables, and independent samples *t* test was used to assess the differences between two groups in continuous variables. Differences between the three surveys were assessed using one-way ANOVA. Univariate general linear models (GLMs) were fitted to estimate the expected means of the SF-36 scale scores with 99% confidence intervals (CIs) for the 2015, 1996, and 2002 surveys and for different respondent ages, adjusted for education and gender. When comparing the different surveys, all respondents under 18 years were removed; this step was only relevant for the 2002 survey which included respondents from 15 years of age (*n* = 155). To assess possible associations between the different SF-36 subscale scores and age, survey year, education and gender, eight multivariable linear regression models were fitted, and the corresponding effect sizes are reported as standardized and unstandardized coefficients. Due to multiple testing, null hypotheses were rejected at significance levels of 1% (*p* < .01). All tests were two-sided. Floor and ceiling effects were considered present if more than 20% of the sample scored the lowest or highest possible score [28]. In the present study, differences in SF-36 subscale scores of 5 points or more were considered clinically relevant [20, 24, 29]. Statistical analyses were performed using IBM SPSS Statistics for Windows, Versions 22.0 and 24.0 (IBM Corp. Armonk, NY).

## Results

### Reference values from the 2015 survey

**Fig. 1:**
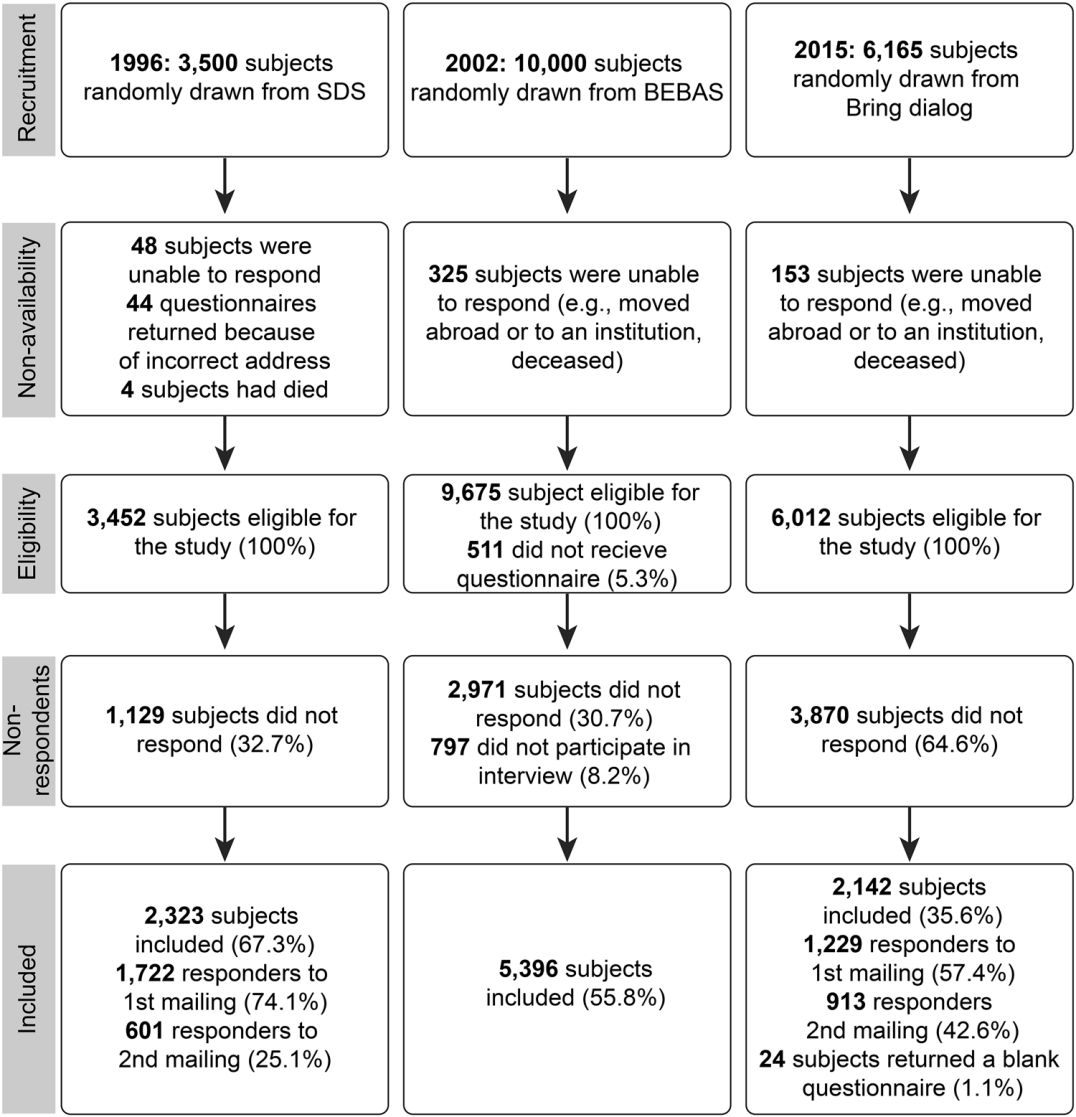
Flowchart of subject inclusion in the three survey

An overview of the inclusion process is presented in Fig. 1. The overall response rate for the 2015 survey was 36%. More females (54%) than males (45%) (*p* < .001) responded, and the responders (55.7 ± 14.1 years) were significantly older than the non-responders (47.7 ± 15.1 years) (*p* < .001). The response rates for both men and women were significantly lower in the youngest age groups (<29 years) than in the older age groups (*p* < .001) (Table 1).

**Table 1:** Basic characteristics, responders, and non-responders in 2015

Variables	Responders (*N* = 2118)	Non-responders (*N* = 3870)	*p*
Age, mean (±SD)	55.7 (±14.1)	47.7 (±15.1)	<.001*
Age groups, *N* (%)			<.001**
≤29 years	105 (5.0)	492 (12.7)	
30–39 years	203 (9.6)	806 (20.8)	
40–49 years	403 (19.0)	873 (22.6)	
50–59 years	484 (22.9)	738 (19.1)	
60–69 years	525 (24.8)	581 (15.0)	
≥70 years	398 (18.8)	380 (9.8)	
Gender, *N* (%)			
Female	1149 (54.2)	1862 (48.1)	<.001**
Male	947 (44.7)	1928 (49.8)	

* Independent sample t test ** *X* ^2^ test *Missing data: [*N*, (%)]: Respondents: Gender: *N* = 22, (1). Non-responders: Gender: *N* = 80 (2.1)

One percent (*n* = 24) of the questionnaires were returned blank. Missing values on the SF-36 ranged from 0.2% (*n* = 5, BP items 21 and 22) to 4% (*n* = 85, GH item 35). Five of the SF-36 scales, PF, RP, BP, SF, and RE had a ceiling effect [28]. Cronbach’s α ranged from .91 (PF) to .79 (MH), indicating an acceptable to excellent internal consistency (Table 2).

**Table 2:** Reliability estimates from the 2015 survey, Cronbach’s alpha for the SF-36 scales, the percentage of subjects with minimum and maximum scores and correlations between the scales

Scale	No. of items	Cronbach’s alpha	%Min./Max.	1	2	3	4	5	6	7	8
1. PF	10	.91	0.5/33.3*	–							
2. RP	4	.90	15.0/63.7*	.643	–						
3. BP	2	.90	0.9/28.5*	.523	.608	–					
4. GH	5	.81	0.1/5.0	.588	.604	.573	–				
5. VT	4	.83	0.9/2.1	.432	.496	.508	.630	–			
6. SF	2	.85	0.8/60.2*	.455	.513	.451	.573	.610	–		
7. RE	3	.83	5.9/80.2*	.325	.399	.304	.382	.420	.544	–	
8. MH	5	.79	0.01/6.1	.239	.276	.314	.464	.647	.637	.532	–

*PF* physical functioning, *RP* role physical, *BP* bodily pain, *GH* general health, *VT* vitality, *SF* social functioning, *RE* role emotional, *MH* mental health, *Min.* minimum, *Max.* maximum

* Ceiling effect, all correlations were significant (*p* < .001)

The age- and gender-standardized scores for the eight subscales are presented in Table 3. The mean scores decreased with age for all scales except for VT, SF, and MH. Women generally scored slightly lower than men and the gender difference was most pronounced and clinically relevant in the youngest age group (≤29 years), in which women scored 10 points lower than men on the BP and VT scales, and 16 points lower on the RP scale. On the GH subscale, there were only small differences that were not clinically relevant between age groups and gender categories.

**Table 3:** Mean SF-36 scale scores (±SD) in 2015 by gender and age group, *N* = 2118

Scales	Age groups													
	18–29 years		30–39 years		40–49 years		50–59 years		60–69 years		70–80 years		Total	
	W (*n* = 65–68)	M (*n* = 36)	W (*n* = 116–120)	M (*n* = 81)	W (*n* = 227–231)	M (*n* = 164–168)	W (*n* = 253–260)	M (*n* = 209–216)	W (*n* = 247–262)	M (*n* = 250–257)	W (*n* = 189–208)	M (*n* = 181–188)	W (*n* = 1097–1149)	M (*n* = 921–946)
PF	93.2 (±14.7)	97.6 (±6.2)	94.0 (±10.2)	94.8 (±9.6)	90.7 (±16.8)	92.8 (±12.6)	87.9 (±16.1)	90.0 (±16.3)	80.8 (±22.6)	85.7 (±18.4)	71.6 (±26.9)	80.3 (±19.3)	84.9 (±21.0)	88.1 (±17.0)
RP	78.3 (±37.8)	94.4 (±18.0)	87.9 (±26.1)	90.7 (±24.5)	77.8 (±37.5)	86.1 (±29.9)	73.4 (±39.3)	82.5 (±32.7)	71.1 (±40.1)	77.0 (±36.1)	57.0 (±43.8)	63.1 (±41.9)	72.6 (±39.6)	78.9 (±35.3)
BP	72.7 (±27.0)	83.5 (±19.1)	76.0 (±23.0)	78.8 (±24.8)	67.0 (±27.5)	71.7 (±26.3)	66.4 (±25.5)	72.4 (±24.8)	64.2 (±26.8)	71.7 (±25.2)	63.5 (±26.7)	67.8 (±25.9)	66.9 (±26.5)	72.1 (±25.4)
GH	74.8 (±21.3)	75.6 (±21.2)	75.2 (±20.3)	73.5 (±20.2)	75.1 (±22.9)	74.5 (±22.0)	72.8 (±22.8)	74.1 (±20.9)	70.7 (±22.6)	72.9 (±21.9)	69.6 (±22.7)	71.1 (±18.1)	72.6 (±22.5)	73.4 (±20.8)
VT	47.6 (±19.6)	58.3 (±18.7)	54.4 (±19.1)	57.8 (±16.8)	56.3 (±19.5)	58.8 (±18.5)	56.1 (±20.8)	62.2 (±18.5)	60.5 (±20.8)	64.0 (±20.3)	60.3 (±21.0)	63.3 (±18.2)	57.2 (±20.6)	61.9 (±18.9)
SF	80.8 (±25.2)	86.4 (±18.7)	84.9 (±24.3)	90.1 (±19.2)	85.3 (±22.1)	89.4 (±21.0)	85.1 (±21.3)	89.7 (±19.1)	87.6 (±19.4)	90.4 (±18.4)	86.5 (±21.5)	86.1 (±19.5)	85.7 (±21.6)	89.0 (±19.3)
RE	78.6 (±33.6)	79.6 (±35.8)	86.6 (±29.7)	92.5 (±21.0)	88.2 (±28.7)	93.4 (±22.9)	91.6 (±22.0)	91.63 (±23.5)	89.2 (±27.5)	89.9 (±26.1)	82.4 (±33.3)	83.7 (±31.2)	87.4 (±28.6)	89.5 (±26.3)
MH	74.5 (±16.0)	75.5 (±15.6)	75.8 (±15.6)	78.2 (±13.2)	78.6 (±15.9)	80.0 (±12.7)	80.2 (±13.8)	81.5 (±13.6)	82.1 (±14.0)	83.9 (±14.2)	82.5 (±13.8)	84.0 (±13.4)	79.9 (±14.8)	81.9 (±13.8)

*PF* physical functioning, *RP* role physical, *BP* bodily pain, *GH* general health, *VT* vitality, *SF* social functioning, *RE* role emotional, *MH* mental health, *W* women, *M* men

### Comparisons of SF-36 scores in 2015, 1996 and 2002

The response rate in 2015 was approximately half of those found in 1996 and 2002 (36% vs. 67% and 56%, respectively). Statistically significant differences in sample characteristics were found between the three surveys. Specifically, respondents in the 2015 survey had a significantly higher mean age than those in the 1996 and 2002 surveys (*p* < .001), and 45% (*n* = 949) had a university degree in 2015 compared to 28% (*n* = 643) in 1996 and 25% (*n* = 1718) in 2002 (*p* < .001) (Table 4).

**Table 4:** Basic characteristics of the respondents in 1996, 2002, and 2015, *N* = 9682

Variables	1996	2002	2015	*p*
Response rate *N* (%)	2323 (67)	5241 (56)	2118 (36)	
Age (years)				
Mean (±SD)	44.9 (±16.5)	47.4 (±16.9)	55.5 (±14.1)	<.001*
Min.–Max.	19–80	18–96	18–79	
Age groups, *N* (%)				
≤29 years	510 (22.0)	870 (16.6)	105 (5.0)	
30–39 years	487 (21.0)	1016 (19.4)	203 (9.6)	
40–49 years	446 (19.2)	1080 (20.6)	403 (19.0)	
50–59 years	363 (15.6)	980 (18.5)	484 (22.9)	
60–69 years	283 (12.2)	657 (12.9)	525 (24.8)	
≥70 years	234 (10.1)	620 (11.8)	398 (18.8)	
Gender, *N* (%)				
Female	1192 (51.3)	2698 (51.5)	1149 (54.2)	.023**
Male	1131 (48.7)	2543 (48.5)	947 (44.7)	
Education, *N* (%)				
Second level, first stage	621 (27.0)	765 (14.6)	377 (17.9)	<.001**
Second level, second stage	1036 (45.0)	2910 (55.5)	782 (37.1)	
Third level (university college or university)	643 (28.0)	1503 (28.7)	949 (45.0)	
Living situation, *N* (%)				
Alone	–	–	358 (16.9)	
With children	–	–	632 (29.8)	
With other adults	–	–	1127 (53.2)	

Missing data: 1996 [*N*, (%)]: education: 23 (1.0), 2002 [*N*, (%)]: education: 63 (1.2%), 2015 [*N*, (%)]: gender: 22 (1.0), education: 10 (0.5), living situation: 1 (<0.001), * One-way ANOVA ** X^2^

To examine the associations between the SF-36 scale scores and sociodemographic factors in the three samples, eight multivariable linear models were estimated (Table 5). To facilitate reading, the number of age groups was limited to four: 18–29, 30–49, 50–64, and 65–96 years. Overall, relatively minor changes were found in the SF-36 scale scores between the three time points: 2015, 1996 and 2002. The participants in the 2015 survey scored statistically significantly (*p* < .001) higher, indicating better function, on the PF and RE scales than the participants in the 1996 and 2002 surveys. The opposite trend was observed for the GH, BP, and VT scales, i.e., there were statistically significantly (*p* < .001) lower scores in 2015 than in 1996 and 2002. Clinically relevant differences were detected in BP and RE, in which the 2015 respondents scored approximately five points lower and seven points higher, respectively, than the respondents in 1996 and 2002.

**Table 5:** Multivariable regression models and associations between SF-36 scales scores and background variables

	PF			RP			BP			GH			VT			SF			RE			MH		
	(*R* ^2^ = .214)			(*R* ^2^ = .116)			(*R* ^2^ = .070)			(*R* ^2^ = .076)			(*R* ^2^ = .028)			(*R* ^2^ = .026)			(*R* ^2^ = .051)			(*R* ^2^ = .021)		
	*N* = 9430			*N* = 9420			*N* = 9519			*N* = 9228			*N* = 9448			*N* = 9545			*N* = 9325			*N* = 9410		
	B	*β*	*p*	B	*β*	*p*	B	*β*	*p*	B	*β*	*p*	**B**	*β*	*p*	B	*β*	*p*	B	*β*	*p*	B	*β*	*p*
1996 (ref)	–	–	–	–	–	–	–	–	–	–	–	–	–	–	–	–	–	–	–	–	–	–	–	–
2002	−0.91	−.02	.043	−1.32	−.01	.145	−1.76	−.03	.006	−1.80	−.04	.001	0.19	.00	.702	0.19	.00	.718	1.81	.02	.021	0.99	.03	.011
2015	2.41	.05	<.001	2.29	.02	.040	−4.6	−.07	<.001	−2.43	−.04	<.001	−2.0	−.04	.001	1.48	.02	.025	7.40	.09	<.001	0.57	.01	.241
Age groups																								
<29 years	3.23	.05	<.001	4.23	.04	<.001	5.18	.07	<.001	2.36	.03	<.001	−1.84	−.03	.004	0.48	.00	.461	−2.22	−.02	.019	−1.67	−.03	.001
30–49 years (ref)	–	–	–	–	–	–	–	–	–	–	–	–	–	–	–	–	–	–	–	–	–	–	–	–
50–64 years	−6.58	−.14	<.001	−8.36	−.09	<.001	−4.92	−.08	<.001	−5.31	−.10	<.001	1.48	.03	.006	−0.67	−.01	.224	−1.59	−.02	.048	1.80	.05	<.001
65–96 years	−17.35	−.35	<.001	−24.61	−.26	<.001	−6.53	−.10	<.001	−8.71	−.15	<.001	2.18	.04	<.001	−2.99	−.05	<.001	−11.24	−.14	<.001	3.48	.09	<.001
Education						.																		
Second level, first stage (ref)	–	–	–	–	–	–	–	–	–	–	–	–	–	–	–	–	–	–	–	–	–	–	–	–
Second level, second stage	6.81	.17	<.001	8.85	.11	<.001	4.95	.09	<.001	5.64	.12	<.001	3.67	.08	<.001	4.43	.10	<.001	8.25	.13	<.001	3.37	.10	<.001
Third level	11.21	.26	<.001	15.24	.19	<.001	11.47	.20	<.001	10.29	.22	<.001	6.03	.13	<.001	6.94	.15	<.001	11.18	.16	<.001	4.46	.13	<.001
Gender																								
Women (ref)	–	–	–	–	–	–	–	–	–	–	–	–	–	–	–	–		–	–	–	–	–	–	–
Men	4.31	.10	<.001	6.06	.08	<.001	4.7	.09	<.001	1.62	.03	<.001	5.2	.12	<.001	3.26	.07	<.001	4.01	.06	<.001	1.89	.06	<.001

*B* unstandardized beta, *β* standardized beta, *PF* physical functioning, *RP* role physical, *BP* bodily pain, *GH* general health, *VT* vitality, *SF* social functioning, *RE* role emotional, *MH* mental health

Missing: *N*: MH: *N* = 272, VT: *N* = 234, BP: *N* = 163, GH: *N* = 454, SF: *N* = 137, PF: *N* = 252, RP: *N* = 262, RE: *N* = 357

The multivariable regression models (Table 5) showed positive significant associations between high education and all SF-36 scale scores (*p* < .001) and between high age (65–96 years) and the VT and MH scales. High age was negatively associated with all other SF-36 scale scores (*p* < .001). Further investigations with sub-analyses indicated that there were some statistically significant effect modifications between survey year and age of respondents (Table 6). The GH decreased by nine points from 1996 to 2015 (*p* < .01) in the youngest age group, while the VT score decreased by approximately seven points from 2002 to 2015 (*p* < .01), and these differences were clinically relevant. Participants in the 30- to 49-year-old age group scored statistically significantly lower on GH, BP, and VT in 2015 than in 1996 and 2002 (*p* < .01), but these differences were only clinically relevant for GH (1996:7.2 points, 2002: 5.8 points) and BP (1996: 6.9 points, 2002: 5.5 points). For the age group 50–64 years, respondents in 2015 scored statistically significantly higher on PF than the respondents in 2002, but the difference was not clinically relevant. The oldest age group in 2015 scored somewhat higher on all scales except for BP, compared to 1996 and 2002, and the differences were statistical significant and clinically relevant for PF, RP, GH, SF, and RE (Table 6).

**Table 6:** Estimated means of SF-36 scale scores and 99% CIs by age group and survey, adjusted for education and gender, *N* = 9682

Subscales	18–29 years			30–49 years			50–64 years			65–96 years		
	1996	2002	2015	1996	2002	2015	1996	2002	2015	1996	2002	2015
PF, mean	93.0	93.4	92.4	90.3	90.2	90.0	84.7	82.6	85.8*	72.5	69.7	78.7*
99% CI	91.0–95.0	91.7–95.0	87.9–96.9	88.8–91.8	89.1–91.2	88.1–91.8	82.6–86.8	81.4–83.9	84.1–87.5	70.1–75.0	68.2–71.3	76.9–80.4
* N*	502	850	102	911	2062	598	472	1332	697	337	893	674
RP, mean	85.1	86.6	80.6	83.2	81.3	80.3	75.1	72.5	74.6	53.8	52.8	65.8*
99% CI	81.0–89.2	83.3–89.8	71.7–89.5	80.1–86.2	79.2–83.4	76.5–84.1	71.0–79.3	70.0–75.0	71.2–78.1	48.7–58.9	49.8–55.8	62.3–69.2
* N*	499	848	103	905	2062	597	467	1329	698	322	897	675
BP, mean	80.6	80.2	74.2	76.3	74.9	69.1*	72.2	68.6	67.3	68.4	67.0	66.6
99% CI	77.7–83.6	77.9–82.5	67.8–80.6	74.2–78.5	73.4–76.4	66.4–71.8	69.3–75.2	66.8–70.4	64.9–69.8	65.0–71.9	64.8–69.1	64.1–69.1
* N*	505	850	103	920	2064	598	489	1336	702	356	914	682
GH, mean	81.7	79.2	72.9*	79.0	77.6	72.1*	72.2	71.3	71.5	68.3	65.8	70.9*
99% CI	79.3–84.2	77.2–81.1	67.5–78.3	77.2–80.8	76.3–78.8	69.8–74.4	69.7–74.7	69.8–72.8	69.4–73.6	65.2–71.3	63.9–67.6	68.8–73.0
* N*	496	844	100	906	2048	586	456	1311	682	315	844	640
VT, mean	58.0	58.5	50.9*	60.1	60.2	55.6*	60.9	61.5	58.9	60.7	60.4	62.5
99% CI	55.6–60.4	56.6–60.4	45.7–56.1	58.4–61.8	59.0–61.5	53.4–57.8	58.6–63.3	60.1–63.0	56.9–60.9	57.9–63.6	58.7–62.2	60.4–64.5
* N*	504	850	102	922	2062	598	482	1328	702	350	876	672
SF, mean	85.8	88.0	81.1	86.0	86.9	85.4	86.2	85.3	86.8	83.3	81.1	87.5*
99% CI	83.4–88.2	86.0–89.9	76.4–87.1	84.2–87.8	85.6–88.1	83.1–87.6	83.8–88.6	83.8–86.8	84.7–88.8	80.4–86.1	79.3–82.9	85.5–89.6
* N*	506	851	103	928	2067	598	492	1339	702	367	912	680
RE, mean	79.8	85.8	76.5	83.9	86.7	87.1	84.5	83.6	88.6	71.5	69.9	86.4*
99% CI	76.3–83.3	83.1–88.6	68.8–84.2	81.3–86.5	84.8–88.5	83.9–90.4	80.9–88.2	81.5–85.8	85.7–91.6	67.1–75.9	67.2–72.5	83.4–89.4
* N*	497	848	102	899	2052	597	461	1321	698	312	875	663
MH, mean	76.4	77.7	74.3	78.3	79.3	77.3	79.0	81.0	80.6	81.9	81.4	83.4
99% CI	74.6–78.2	76.3–79.1	70.4–78.2	77.0–79.6	78.3–80.2	75.7–79.0	77.2–80.8	79.9–82.1	79.1–82.2	79.7–84.0	80.0–82.7	81.8–84.9
* N*	504	850	103	922	2060	598	479	1328	702	339	859	666

*PF* physical functioning, *RP* role physical, *BP* bodily pain, *GH* general health, *VT* vitality, *SF* social functioning, *RE* role emotional, *MH* mental health

* *p* < .01

## Discussion

This study provides new Norwegian reference values for the SF-36 based on data from 2118 men and women aged 18–80 years collected in 2015. The randomly drawn sample was representative of the general Norwegian population with respect to age, gender, and place of residence. However, only 36% of the sample responded to the survey. Compared to similar surveys in 1996 and 2002 this response rate was low. However, the stability in scores on all HRQoL domains across the three surveys was high indicating a relatively stable HRQoL in the Norwegian population during the past 19 years, although significant changes were found in certain age groups. Interestingly, the older respondents (≥65 years) in 2015 scored higher on all SF-36 scales than the respondents in 1996 and 2002, except for BP.

The 2015 survey was specifically designed to collect updated reference values for the Norwegian version of SF-36v1 as requested by recent research [16]. However, the low response rate in the 2015 survey questions the representativity of the collected reference values and there are some discrepancies when comparing the sample to the actual composition of the Norwegian population in 2015. About 21% of the population was between 18 and 29 years, while only 5% of this age group participated in the survey. For the older part of population, the opposite pattern was seen. Eighteen percent was 67 years or above, while 27% of the responders were in the same age group [30]. Both findings suggest that the reference values are not fully representative for the Norwegian population with respect to age. Another factor that reduces the representativity is the large proportion of the 2015 sample with a university or college university education. According to Statistics Norway 32% of the population had a higher education in 2015, 41% had finished high school, and 27% had only finished elementary school. In the 2015 sample 45% had higher education, 37% had finished high school, and 18% had finished the lowest education level [31]. These findings bear out two important points. Firstly, the reference values from 2015 should be used with care when performing comparisons for the younger population and for subjects with low education. Secondly, the reference values from 2015 can be assumed to describe the HRQoL in older people better than the 1996 sample since that study had a relatively low response rate for the older parts of the population [14]. The decline in response rate from 67% in 1996 to 36% in 2015 is in accordance with both national [32] and international [33–35] findings regarding response rates to postal surveys in the past 15 years. Multiple factors may affect response rates such as the length of the survey, use of pre-notifications, follow-up contact, and survey mode [36]. The same method was used in the three surveys: distribution by mail. Given the digital era of today, one may wonder if the response rate would have been better with an electronic survey. However, some studies have concluded that the use of electronic surveys has a comparable [37] or even lower response rate compared to other survey modes [38]. Suggested explanations have been lack of internet access or computer experience. In 2015, 97% of all households (with at least one person aged <75 years) in Norway had access to the internet [39]. Thus, lack of internet access would therefore probably not have been a challenge in Norway. Also, a study showed that despite having internet access and experience using it, respondents chose to reply on paper rather that online. This may be caused by a fear of sending sensitive personal data over the internet [40]. SF-36 clearly has questions of a sensitive nature, so it is not given that the use of electronic surveys would have increased the response rate in the present surveys. Thus, the external validity of the 2015 data may have been compromised by a potential non-response bias [34]. However, some studies have suggested that higher response rates would not provide different results [23, 33, 41, 42]. Furthermore, a Norwegian study found that HRQoL measured with EORTC QLQ-C30 was relatively stable in two cross-sectional studies eight years apart, despite the fact that the response rate was 33 percentage points higher in the first study than in the second (68% vs. 35%, respectively) [23]. Other studies have suggested that although the estimates may change when including non-responders, the associations may not significantly differ [43, 44].

The scores for the PF, RP, BP, SF, and RE scales showed an extensive ceiling effect. Studies investigating self-perceived health often struggle with ceiling effects [45], and similar results have been reported in previous studies [14, 20]. The ceiling effects detected in the 2015 survey is comparable to the ones reported in 1996 [14] and in 2002 [16]. The biggest differences are an increase of 8.9 percentage points on the RE scale (1996: 71.3% max score vs. 2015: 80.2% max score) and a reduction of 5.6 percentage points on the GH scale (1996: 10.6% max score vs. 2015: 5.0% max score). A possible explanation of the ceiling effect may be the inclusion of a non-hospitalized sample in which scores on the PF, RP, SF, and RE scales are expected to be high. However, it may also suggest that the subjects with the highest burden of disease do not respond, and that the reference values therefore may be biased since results from the sickest individuals are lacking. Still, in comparison with diseased populations, floor effects are of greater concern since these might camouflage differences of clinical importance.

The high proportion of respondents over 70 years may reflect the increased life expectancy in Norway and that elderly who are fit constitute a larger proportion of this age group [46]. The life expectancy at birth in 1996 for women was 81.0 years, compared to 84.1 years in 2015. An even larger improvement is found for men, from 75.3 in 1996 to 80.3 in 2015 [47]. In 2015, the oldest age group scored higher on all SF-36 scale (except BP) compared to these groups in 1996 and 2002. This result can be explained by several factors. First, there has been an increase in healthy life years in the Norwegian population, and in general, eight out of 10 Norwegians report that they have good to very good health [17]. Second, focusing on health promotion and preventing functional decline in community-dwelling elderly are important goals of the Norwegian government, and thus multiple measures have been implemented to attain this goal [48]. However, the higher scores may also represent a healthy bias in the elderly [14]. The elderly with the lowest HRQoL and perhaps the highest burden of disease probably did not participate.

Even if the older age group in 2015 scored higher than in 1996 and 2002, still all physical subscales were negatively affected by increasing age, which is also consistent with previous national [14] and international results [20, 22, 27]. The reduction in physical function may be related to both increased morbidity and the biological aging process which are known to influence physical function through effects such as decline in maximal aerobic capacity, reduced skeletal muscle performance, and changing body composition [49, 50]. For the VT, SF, and MH subscales the opposite trend was observed. The oldest age groups scored higher than the youngest age group who reported the lowest scores on these scales. Previous studies have found similar results of higher VT [14, 27], SF [27], and MH [14, 27] scores in older respondents. Several studies have reported increasing life satisfaction and subjective well-being in older individuals [51, 52]. Perceived subjective well-being and life satisfaction may remain high despite morbidity and/or advanced age. Over time, individuals suffering from chronic conditions or those of advanced age may grow accustomed to their situation [29], and their subjective life expectations may change. An important mediator of this physiological process is “response shift”, which involves adapting and adjusting one’s internal standards, goals, values, and conceptualizations underlying reports of HRQoL [53, 54].

Our results show that education is an important predictor of HRQoL in the Norwegian population. Even though a significantly larger proportion of the respondents in 2015 had completed university/university college education compared to the corresponding proportion in 1996 and 2002, HRQoL remained relatively stable. One explanation for this result may be the absence of potentially important variables in the regression models such as medical conditions [55], lifestyle behavior [56], income [57], and employment status [58]. In previous studies, these factors have been shown to significantly affect HRQoL and may explain why HRQoL did not increase despite the higher education level in the population in 2015.

An important limitation in the present study is the difference in the sample selection of the three surveys. First, both the 2015 and 1996 surveys were designed to collect normative data for SF-36, whereas the 2002 survey was part of an annual cross-sectional study investigating the living conditions in Norway. Second, the SF-36 data from the 2002 survey were part of a larger survey that included telephone or home interview with the respondents regarding health status, presence of chronic diseases, etc. before they received the postal survey containing SF-36 [15]. The respondents in the 2015 and 1996 surveys had no contact with the researchers or other study personnel. Considering that the 2002 survey respondents had contact with the interviewers in advance, this might have affected the motivation for filling out the questionnaire, thus influencing the response rate [16], most probably increasing it somewhat. Third, the layouts and designs of the questionnaires were slightly different in the three different surveys. Even though, the SF-36 questions were identical, one can never rule out that different layouts may have affected the response rates [16]. Another limitation is that the samples’ basic characteristics are statistically significantly different in terms of age, gender, and education level. These differences between samples may have affected the response rates and thus our results. Further, as previously discussed, some differences between the samples were expected a priory, given the demographic changes in the general Norwegian population during the past 19 years, i.e., longer life expectancy, higher levels of education, and maybe larger difference between the very active and healthy on the one hand and the sedentary on the other.

Our overall finding is that relatively minor changes in HRQoL assessed by the SF-36 appear in the Norwegian population over a period of 19 years. This is consistent with findings from other studies, with both cross-sectional and prospective designs [20, 22]. The most pronounced differences were found in the youngest and oldest age groups. This may be interpreted as a result of certain demographic changes, e.g., a longer life expectancy and better health among the oldest. It may also be attributed to a healthy bias in this group and a response bias in the youngest age group.

## Conclusion

From a practical standpoint, the present study provided updated Norwegian reference values on the SF-36 v1, which can be used as an anchor point for comparisons with other samples in research and clinical practice. The low response rate, and thus the questionable representativity in the 2015 survey, suggest that the reference values, especially for the youngest age group, should be used and interpreted with caution. The response rate in the oldest age group was high, and the revised reference values can likely be used for people aged >70 years.

Despite the significant changes in Norwegian society over the past two decades, HRQoL has remained relatively stable, hence societal changes may not have affected HRQoL as much as expected. To increase the response rate in future studies, data collection by electronic surveys should be considered, due to the high internet access in Norway and increasing computer experience in the population.
